# Rapid range shifts in African *Anopheles* mosquitoes over the last century

**DOI:** 10.1098/rsbl.2022.0365

**Published:** 2023-02-15

**Authors:** Colin J. Carlson, Ellen Bannon, Emily Mendenhall, Timothy Newfield, Shweta Bansal

**Affiliations:** ^1^ Department of Biology, Georgetown University, Washington, DC 20057, USA; ^2^ Science, Technology, and International Affairs Program, Edmund A. Walsh School of Foreign Service, Georgetown University, Washington, DC 20057, USA; ^3^ Department of History, Georgetown University, Washington, DC 20057, USA

**Keywords:** *Anopheles*, malaria, geographical range shift, climate change

## Abstract

Facing a warming climate, many tropical species—including the arthropod vectors of several infectious diseases—will be displaced to higher latitudes and elevations. These shifts are frequently projected for the future, but rarely documented in the present day. Here, we use one of the most comprehensive datasets ever compiled by medical entomologists to track the observed range limits of African malaria mosquito vectors (*Anopheles* spp.) from 1898 to 2016. Using a simple regression approach, we estimate that these species’ ranges gained an average of 6.5 m of elevation per year, and the southern limits of their ranges moved polewards 4.7 km per year. These shifts would be consistent with the local velocity of recent climate change, and might help explain the incursion of malaria transmission into new areas over the past few decades. Confirming that climate change underlies these shifts, and applying similar methods to other disease vectors, are important directions for future research.

## 1. Introduction

In the coming century, most scientific evidence projects that climate change will be responsible for a massive redistribution of global biodiversity [1–3]. Today, the world is already +1.2°C warmer than in the pre-industrial period [4], and this transition is already underway: tropical species are spreading towards the poles, and species everywhere are tracking their thermal niche along elevational gradients. One foundational meta-analysis estimated that, to date, terrestrial species have been moving uphill at a pace of 1.1 m per year, and to higher latitudes at a pace of 1.7 km per year [5].

Among the millions of species on the move are some of the most consequential pathogens, disease vectors and wildlife reservoirs that affect human health and economic development. For example, one study estimated that crop pathogens and agricultural pests were undergoing latitudinal shifts of 3 km per year [6]. Similarly, the North American vector of Lyme disease, the deer tick *Ixodes scapularis*, has spread over 40 km per year in the northeast [7,8]; the northern and elevational range limits of *Ix. ricinus* ticks have expanded similarly rapidly in Europe [9–11]. In recent years, mosquito-borne diseases like malaria, dengue and Zika virus have also expanded to new latitudes and elevations [12–14], and will continue to do so in the future, following the thermal limits on transmission set by their ectothermic vectors [15–17]. Some of these expansions have been facilitated by parallel global invasions of *Aedes aegypti* and *Ae. albopictus*, which have spread an estimated 250 and 150 km per year, respectively; climate change will allow their spread to continue over the coming century, albeit at a slower pace [18,19].

However, surprisingly little is known about the impacts of climate change on the anopheline vectors of malaria, lymphatic filariasis and O'nyong'nyong virus. Already, warming temperatures could have plausibly permitted expansions into highland east Africa [20]*;* some *Anopheles* species have become newly established in high-elevation sites in Latin America [21]; and a groundbreaking study recently found that in the Sahel, these mosquitoes can migrate hundreds of kilometres overnight, transported by wind currents [22]; but no studies have examined whether range shifts are already underway in these species. Here, we revisit a comprehensive dataset describing the geographical distributions of the primary malaria vectors (*Anopheles* spp.) in sub-Saharan Africa, and test the idea that over the last century, these species have moved southward (away from the equator) and upward (gaining elevation), consistent with hypothesized climate impacts.

## 2. Methods

### (a) Data

We revisit a recently published compendium of occurrence data for 22 species of *Anopheles* mosquitoes vectors of malaria in Africa [23]. While some of these data are resolved to finer taxonomy, we used the broadest possible definitions at the species level, treating *Anopheles funestus sensu lato* and *sensu stricto* as one species, and all members of the *Anopheles gambiae* complex—including *An. gambiae s.l., s.s.,* M form, and S form—as another single species. (We elect not to stratify these further, given that some are a nested subset of others, in ways that would be challenging to develop *ad hoc* rules around.)

**Figure 1.**
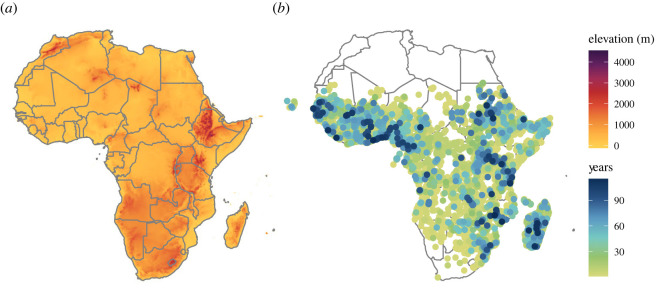
(*a*) The elevational gradient in Africa (averaged to a 10 × 10 fold higher resolution for visual clarity; (*b*) sites of *Anopheles* mosquito occurrence in sub-Saharan Africa, where colour represents the maximum temporal span of observations.

In total, the dataset comprises over a century's (1898–2016) worth of long-term, systematic entomological surveys from malaria programmes, as well as other opportunistic data collected by researchers, gathered from a mix of peer-reviewed publications, technical reports, theses, and archival records. Records span more than 1 year at the majority of sampling sites (61%), covering an average of 8.5 years between the first and last presence record (figure 1*b*). Parsed into unique spatio-temporal records, the dataset includes a total of 5 04 314 year-locality pairs, with an average of 22 923 records per species. While sampling fluctuates over time and increases during the Global Malaria Eradication Program (GMEP; 1955–1969), the dataset spans the entire century with an incredible level of detail (electronic supplementary material, figure S1).

For elevational data, we used the GTOPO30 global digital elevation model downloaded as a 30 arc-second resolution grid for Africa from Data Basin (www.databasin.org; figure 1*a*). We extracted elevation for each distinct occurrence record, using the ‘raster' package in R version 3.3.2. In each year, we extracted the highest-elevation and southernmost records by species. A total of 116 unique locality points, situated on coastlines or islands (e.g. Cape Verde), were beyond the spatial extent of the raster and so were not assigned elevational data.

### (b) Analysis

Studies that analyse evidence for geographical range shifts tend to fall into two categories: (a) simpler study designs that measure change in observed range maxima over time, or (b) more complex designs that use an explicit model of the observation process to estimate the true range boundaries, often using data from throughout the entire geographical range. Our study falls into the first category, building on studies that use ‘pre-post' analyses of range boundaries during multiple discrete intervals [5,24]. These studies generally use a Mann–Whitney test or a similar non-parametric approach to test for significance, and estimate the pace of an observed shift in range boundaries by interpolating between two timepoints. Here, we expand on that approach by using a simple linear regression to both (a) measure the rate at which the observed maxima have changed through continuous time, and (b) apply a built-in measure of significance to the temporal trend. Our approach is only a descriptive tool for summarizing change in the data, and does not estimate true range boundaries or identify causal factors.

For each of 22 species of *Anopheles*, we used a simple linear regression with the ‘stats’ R package to estimate change in the maximum recorded elevation *e* and southernmost latitude *l* for species *i*, based on values derived from the maximum in each year *t* since the first record for that species:

2.1 $$e_{t,i}\;=\beta_{0,i}+\beta_{1,i}t$$

and

2.2 $$l_{t,i}\;=\beta_{2,i}+\beta_{3,i}t.$$

The slopes of these regressions *β*_1,*i*_ and *β*_3,*i*_ were taken as the estimates of range shift velocity for species *i*. We limited this analysis to cases with at least five or more unique values over time: with this cut-off, we were able to estimate latitudinal trends for 18 species, elevational trends for 20 species, and both for a total of 17 species. Species-level estimates are given in electronic supplementary material, table S1 and other regression summary statistics are given in electronic supplementary material, table S2.

### (c) Limitations

Our dataset includes several kinds of unaddressed heterogeneity, including a century of changes in sampling methodology, species identification (including the rise of molecular methods), and malaria monitoring and control. We anticipate few of these would have a directional effect on our estimates, with the notable exception of an overall trend towards greater sampling intensity. For all modelled species except the rarest in the dataset (*An. bwambae*), the number of observations has a strong temporal trend (*p* < 0.001). This could create an artificial bias towards observing false expansions (i.e. higher extreme values may be detected in progressively larger samples drawn from the same underlying observation process).

As a preliminary sensitivity analysis, we ran a second set of models that included the number of observations (by species, per year) as a second predictor

2.3 $$e_{t,i}\;=\beta_{0,i}+\beta_{1,i}t+\beta_{2,i}n_{t,i}$$

and

2.4 $$l_{t,i}\;=\beta_{3,i}+\beta_{4,i}t+\beta_{5,i}n_{t,i}.$$

This approach should be interpreted with caution, as it only evaluates whether a temporal trend in range margins is still detectable from a relatively small number of unique data points (approx. 120 or fewer) after accounting for a tightly collinear temporal trend in sampling. Other methods have been proposed that go further, and account for sampling intensity by explicitly estimating ‘true' range boundaries [25,26]. For the most part, these are better suited for smaller, simpler landscapes; a small number of approaches exist that would be able to demarcate species ranges that span the African continent (e.g. [27]), but they are orders of magnitude more computationally intensive, and should be explored in future work.

Our modelling approach also fails to capture any potential nonlinearity in long-term trends. Over the interval of observation, the rates of warming and other environmental changes have been non-stationary, and might not have reached biologically meaningful levels until the latter half of the century [28]. In addition, we expect there might be both lags between climatic changes and range shifts, and additional nonlinearities unrelated to climate in the range shift process, particularly as a result of vector control efforts. Empirical evidence for either pattern is sparse in the broader biological literature, and methodological frameworks to accommodate both are limited. In future work, some of the approaches that better address sampling limitations and climate attribution could also readily improve handling of nonlinear temporal dynamics [27].

## 3. Results

**Figure 2.**
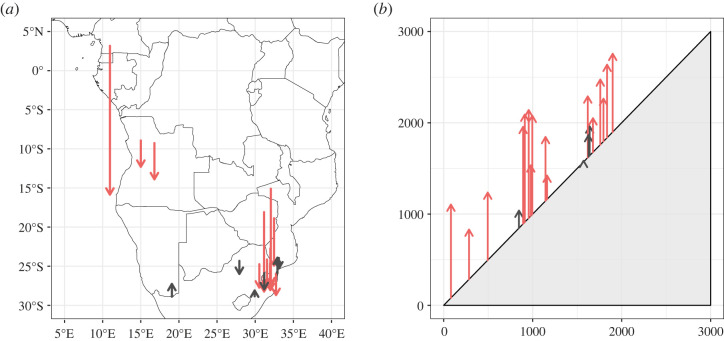
Estimated shifts in *Anopheles* species’ observed latitudinal and elevational maxima over the twentieth century, where each arrow gives one species’ estimate (see electronic supplementary material, table S1). (*a*) Species’ estimated southern maxima, where starting points are given at the longitude of the southmost point in the first half of the century (1900–1950), and the arrow shows the estimated latitudinal shift from 1900 to 2000 (chosen as a standardized unit for visualization, rather than the entire observation period, given that some species are sampled over slightly different intervals). (*b*) Estimated elevational gain, 1900–2000 (*y*-axis), on a 1:1 elevational ‘gradient' (*x*-axis gives initial estimated elevational position). Red arrows indicate species for which temporal trends were statistically significant (*p* < 0.05).

In both elevational and latitudinal maxima, we observed a strong and unambiguous signal consistent with long-term range expansion (figure 2). We found that species’ southern range maxima shifted at an estimated pace of 4.7 km each year (± s.d. of 5.7 km), with 16 of 18 species exhibiting a significant trend (cut-off of *p* < 0.05). Elevational maxima also shifted rapidly, at an average estimated rate of 6.5 m of altitudinal gain per year (± 3.5 m), with 18 of 20 species exhibiting a significant trend. All estimated elevational trends and most latitudinal trends (15 of 18) were positive, i.e. were consistent with the direction expected from climate-linked geographical range shifts. The correlation between species’ estimated velocity of elevational shifts and estimated velocity of latitudinal shifts (*r* = 0.34) was insignificant (*p* = 0.19), suggesting that landscape-level patterns had a stronger influence on the pace of range shifts than variation among species’ intrinsic capacity for dispersal.

Finally, the sensitivity analysis revealed that observed range maxima were responsive to sampling size, but with different implications for the two variables. Including sampling reduced the rate of observed shifts (though they remained directionally consistent with expectations) to an average latitudinal gain of 2.38 ± 1.66 km per year and an elevational gain of 1.90 ± 0.55 m per year. The effect of sampling was only significant (at *p* < 0.05) in 12 of 18 models of latitude, and 14 of 18 temporal trends in latitude remained significant. However, for elevation, all 20 models recovered a significant effect of sampling, and only six species still showed a significant trend over time. These results suggest that observed shifts are unlikely to be artefactual, but that sampling effort contributes to observed trends, and may be a more serious confounder over the smaller landscapes that are relevant to elevational gradients.

## 4. Discussion

We found clear evidence that *Anopheles* mosquitoes have undergone rapid range shifts over the twentieth century, challenging a long-standing assumption in historical epidemiology that mosquito ranges are mostly stationary over decades or centuries [29,30]. Our findings were consistent with expectations for the direction and pace of climate-linked range shifts, including previous estimates of climate velocity in sub-Saharan Africa [31]. Future work could build on these findings by using more sophisticated methods, such as spatio-temporal occupancy models [27], to formally test the explanatory power of climate change while accounting for sampling bias. If confirmed, the rapid expansion of *Anopheles* ranges—on average, over 500 km southward and 700 m uphill during the period of observation—would rank among the more consequential climate change impacts on African biodiversity that have been observed to date.

These findings could also suggest a new facet of the complex and contentious relationship between climate change and shifting malaria endemicity in Africa. The thermal limits of the *Plasmodium* parasite are well established [32,33], and readily superimposed onto climate projections. This mechanistic approach has suggested that malaria will spread into highland east Africa and expand at its southern limits, but transmission will likely decrease as west and central Africa become prohibitively warm [17,34]. Beginning in the early 2000s, several studies have proposed that these impacts might already be observable in east Africa [35–37]. Others have disputed these conclusions, suggesting that they are irreconcilable with long-term progress towards malaria elimination, that trends in the region are better explained by lapsed control programmes and growing drug resistance [38–41], and that climate change is inconsistent with long-term trends at the continental scale [42]. These debates—which remain unresolved—have focused nearly entirely on *P. falciparum* prevalence or incidence, and have rarely considered direct impacts of climate change on the mosquito vectors of the parasite.

If climate change has allowed *Anopheles* mosquitoes to invade once-protected colder areas, this might help explain observed changes in the altitudinal limits of malaria transmission [13], without presuming the veracity of a climate-driven, long-term increase in prevalence in these areas. Confirming this chain of causation would be an important step towards resolving one of the longest-standing debates in climate and health research. More broadly, in the coming years, these sorts of direct links between climate, biodiversity change and disease emergence will be increasingly important to quantify in real-time, not just to document a changing world but also to identify and address healthcare needs in newly vulnerable populations.

## Data Availability

No original data are used in this study. The *Anopheles* dataset is freely available from other previously published research [23]. Electronic supplementary material is available online [43].
